# “I'm Managing My Diabetes between Two Worlds”: Beliefs and Experiences of Diabetes Management in British South Asians on Holiday in the East—A Qualitative Study

**DOI:** 10.1155/2016/5436174

**Published:** 2015-11-30

**Authors:** Neesha R. Patel, Anne Kennedy, Christian Blickem, David Reeves, Carolyn Chew-Graham

**Affiliations:** ^1^Centre for Endocrinology and Diabetes and Manchester Centre for Health Psychology, Institute of Human Development, The University of Manchester, Room S42, Second Floor, Zochonis Building, Brunswick Street, Manchester M13 9PT, UK; ^2^NIHR Collaboration for Leadership in Applied Health Research and Care (CLAHRC) Wessex, Faculty of Health Sciences, University of Southampton, Highfield Campus, Building 67, Southampton SO17 1BJ, UK; ^3^NIHR Collaboration for Leadership in Applied Health Research and Care (CLAHRC) Greater Manchester, Centre for Primary Care, 5th Floor, Williamson Building, Oxford Road, Manchester M13 9PL, UK; ^4^Centre for Primary Care, 5th Floor, Williamson Building, Oxford Road, Manchester M13 9PL, UK; ^5^Research Institute, Primary Care and Health Sciences and NIHR Collaboration for Leadership in Applied Health Research and Care (CLAHRC) West Midlands, Keele University, Keele, Staffordshire ST5 5BG, UK

## Abstract

*Background*. Diabetes is disproportionately high among British South Asians compared to the general UK population. Whilst the migrant British South Asians group has received most attention on research related to diabetes management, little consideration has been given to impact of travel back to the East. This study aimed to explore the role of social networks and beliefs about diabetes in British South Asians, to better understand their management behaviours whilst holidaying in the East. *Methods*. Semistructured interviews were conducted in Greater Manchester. Forty-four participants were recruited using random and purposive sampling techniques. Interviews were analysed thematically using a constant comparison approach. *Results*. Migrant British South Asians expressed a strong preference to be in a hot climate; they felt they had a healthier lifestyle in the East and often altered or abandoned their diabetes medication. Information acquisition on diabetes and availability of social networks in the East was valued. *Conclusion*. Social networks in the East are a valued source of information and support for diabetes. The lack of adherence to medication whilst abroad suggests that some migrant British South Asians have a poor understanding of diabetes. Future research needs to explore whether patients are seeking professional advice on diabetes management prior to their extended holiday.

## 1. Introduction

Diabetes is a life-long chronic and progressive condition affecting 3.2 million people in the UK, and 90% of people have type 2 diabetes (T2D) [1]. T2D and its associated complications are disproportionately high among British South Asians compared to the general UK population [2]. The onset of T2D is related to genetic predisposition, poor diet, obesity, and physical inactivity; additional factors such as cultural health beliefs, language difficulties, and access to healthcare service have also been suggested to influence the higher incidence of T2D in British South Asians [3]. Migration from the Indian subcontinent (e.g., Indian, Pakistani, Bangladeshi, and Sri Lankan) has been associated with the onset of T2D in British South Asians due to changes in lifestyle and diet [4–6] as well as the stresses of adapting to the UK and the emotional upheaval of leaving the Indian subcontinent [7, 8].

Diabetes self-management is vital and has been stated as one of the most challenging regimes of any chronic illness due to the extensive number of tasks involved in managing blood sugar levels and reducing the risks of serious complication including hypertension, stroke, kidney failure, heart disease, and neuropathy [3]. However, studies with migrant British South Asians have found this group to have poor knowledge and understanding of the seriousness of diabetes [9], lower perceived awareness of its complications, poor knowledge about diet, and poor adherence to medication [10] resulting in poor diabetes outcomes [11]. Similar findings in terms of knowledge and attitudes of diabetes have also been reported in South Asians residing in the East [12, 13].

Culturally sensitive diabetes education programmes designed to improve self-management in this population have had limited success in improving diabetes outcomes [14]. Whilst the migrant British South Asian group has received most attention in the literature on research related to incidence of diabetes and its management [15], very little consideration has been given to the effects of migration on diabetes management in this population [4], particularly with regard to whether their beliefs and behaviours concerning diabetes change when they travel back to the Indian subcontinent for extended holidays. The need to provide education to British South Asians on aspects of travel abroad and adhering to medication was outlined in a review by Hawthorne et al. (1993) [16]. This is potentially important as it is common for migrant British South Asians in the UK to travel to the East regularly, especially during the UK winter months to escape the cold weather. Thus, there is a possibility that people make changes to their diabetes regimen during their stay in the East and may not be aware of the importance of continuing to manage their diabetes whilst travelling and holidaying in the Indian subcontinent. In addition, people will often stay with family or friends in the East; therefore, it is also important to consider the changes in people's social context for diabetes management, as there is an increasing recognition that social networks (e.g., strong family ties and friends) contribute to diabetes management as well as providing practical and emotional support to the work individuals with diabetes undertake for their diabetes [17, 18]. Social networks also have the potential to shape beliefs, attitudes, and information acquisition for diabetes [19–22] and it is likely that management practices and lifestyle behaviours whilst holidaying in the East may be influenced by this context.

To date, the advice on travelling and diabetes provides general information on the precautions people should take in terms of diet and medication supplies [23] and the impact of jet lag, time zone differences, which may affect adherence to medication and thus blood glucose levels [24]. However, there is lack of advice for British South Asians with diabetes on the importance of adhering to the diabetes regimen when holidaying in the East for an extended period of time, and it is not clear whether patients actually consult or seek advice from their GP or Practice Nurse (PN) about their intentions and/or plans to travel abroad for long periods of time. This could usefully be addressed in consultation with a GP and/or PN as most patients are managed and supported by primary care in the UK.

In the study reported here, we exploreself-reported beliefs and practices of diabetes management in British South Asians, to better understand their management behaviours whilst holidaying in the East. Data was collected within the context of a broader study around diabetes management and social networks.

## 2. Methods

This study was conducted as part of National Institute for Health Research (NIHR) Collaboration for Leadership in Applied Health Research and Care (CLAHRC), Long Term Conditions (LTC) programme, and ethical approval was granted through this programme of research (Reference 10/H1008/1 09130).

British South Asian adults with type 1 diabetes (T1D) or T2D, living in Greater Manchester, were recruited using two methods of sampling; 30 participants were recruited using random sampling of 22 GP registers and additional 14 participants were recruited using purposive sampling to obtain a broader sample of participants from community groups (mosques, temples, religious classes, exercise groups, and Muslim day centres). Interviews were conducted with participants in a location of their choice, mainly their own homes.

Semistructured face-to-face interviews were conducted with participants between March 2010 and July 2011. The interviews lasted between 30 and 90 minutes and were audio recorded with consent. The length of an interview is known to vary depending on the topic, researcher, and participant [25]. A topic guide was developed by the gaps identified from the literature in this field (as mentioned in the Introduction) and through discussion with the research team to explore a range of beliefs and practices concerning diabetes management including fasting, diet, and use of self-management resources, medication, and support from social networks.

Data collection and analysis were iterative with modification of the topic guide as analysis progressed.

One interview was conducted in Hindi by the first author (Neesha Patel). A professional interpreter, independent of the project, provided language support for Urdu speaking respondents whose first language was not English (*n* = 9). On other occasions, where this was requested, members of patients' families sometimes helped with interpretation. In two interviews, a Diabetes Asian Link worker was present to provide language support. All respondents were reimbursed £15 for their time.

## 3. Data Analysis

Initially open coding was used to analyse the transcripts and, through comparison of these codes, categories and themes were identified. Thereafter, data were analysed thematically using a constant comparison approach [26]. Themes were developed independently by all authors and then agreed on through discussion. Field notes and written memos were used to help develop interpretations during analysis. Data collection was continued until category saturation was achieved in that interviews continued until no new themes emerged from the data. Atlas.ti6 software was used to store and manage the data.

## 4. Results

Forty-four people were interviewed.  Table 1 shows the demographic characteristics of the participants studied. The majority of South Asian participants with T2D in this study were migrants from the Indian subcontinent. Thus, the data in this section relates mainly to this group, along with reference to UK born South Asians where appropriate.

Data is presented in three main themes: social networks,* differing roles and opportunities for social support from networks* “back home,” beliefs about diet and diabetes management, and limited role for GP/practice.

Data is presented to illustrate the themes, and participants are identified by their diabetes type, gender, and method of recruitment with an asterisk to indicate a participant has been quoted more than once.

### 4.1. Social Networks, Differing Roles and Opportunities for Social Support from Networks “Back Home”

The availability of family members in the East seemed to have an important role in providing more care and facilitating dietary behaviours.

*She can't take care of herself much down here [UK] because my brother is always at work, I am at my place, my sisters are always at college or busy so she is always on her own but Pakistan she has her dad, brothers and sisters and their kids. [Community participant 6, Pakistani, female, T2D]*

*P: When I came back from Pakistan I was healthier than I was here (in the UK) and I felt much better because my sister-in law goes to the park for a walk for 2 hours…when she comes home she will eat. I copied her and I felt much better. [Participant 326, Pakistani, female, T2D]*

The families abroad were also an important source of information for diabetes and would often provide information on diet and foods, and participants seem to value and follow the advice, which was believed to be beneficial to diabetes:

*lots of people in the family have it so they used to tell me try this and that to reduce my sugar levels, e.g. karela juice but it's very bitter and all day I can taste it in my throat which I didn't like. (Community participant 27, Pakistani, female, T2D)*

*I: Do people give advice you advice on how to manage diabetes?*

*P: Yeah that's always in the family*

*I: It is different type of advice in Pakistan compared to the advice you get from people here [in the UK)?*

*P: Yeah it's the same advice like; they tell you what's good for you and what's not for diabetes. Like sometimes you learn about foods that are good for your diabetes, like certain vegetables. [Participant 81, Pakistani, male, T2D]*

In addition to receiving information and advice from family members in the East, some participants also described receiving advice from external sources in the UK.

*I: When you're there (in the East) do people give you advice on your diabetes? *

*P: Some of my friends know about diabetes and if they are not around then I go to the doctors.*

*I: What kind of things do they tell you to do?*

*P: To be careful about the sweet things that I have and diet.*

*I: So what about here in the UK do people tell you that here?*

*P: No no-one in the family but Asian Link Worker, doctor and nurses tell me and when I go to the mosque to pray other people that have diabetes they talk about it and they all give different reasons. (Community participant 5, Bangladeshi, Male, T2D)*

### 4.2. Beliefs about Diet and Diabetes Management

Compared to UK born South Asians, it was more common for migrant British South Asians to travel “back home” (India, Pakistan, or Bangladesh) for a prolonged period of time (six to eight weeks or more). However, going “back home” was reported to have a positive influence on diabetes management, mainly attributed to the healing effects of being with family and the hot weather conditions in the East.

Many of the migrant British South Asian participants with T2D believed that their diet was much healthier “back home” due to the availability and daily consumption of more fruit and vegetables.

*P: You get fresh fruit and vegetables every day, they come to the house with a cart every day or there are markets nearby too but it's fresh every day. (Participant 401, Indian, male, T2D)*

The foods in the East were also believed to be fresher and easier to access compared to the West, where participants often described using frozen foods rather than going to the supermarket every day to buy fresh foods.

*Daughter: Here you cannot get fresh vegetables; there [Pakistan] you can so she has fresh stuff all the time. Here whatever is in the freezer she will take it out and cook it? She gets a lot of fresh fruit and vegetables from there. So there is a big difference with how she deals with things here and how she does it there. There she is healthier so she is active down there, fresh fruit is always better, so she takes care of herself down there. *

Participants described walking more with family members and adapting to their family's comparatively healthier lifestyle whilst on holiday in the East, compared to when they are back in the UK.

*P: You can't get out and enjoy and you don't have the freedom to go out and do things like go for walks for a start but err everybody gets a bit low in the winter times…I would like to live in India for 6 months and here for 6 months. [Participant 7, Indian male, T2D]*

Holidaying “back home” seemed to give a sense of freedom and motivation to engage in healthy behaviours and live a healthy lifestyle.

*P: Whenever I go India…you feel like going out and you're not restricted to do anything, whereas, as soon as you come here, you're in front of the box (TV) twenty four seven and that's your life now. [Participant 332, Indian, male, T2D]*

*P: Here (in the UK) most of the time its damp and raining we stay indoors and do not move much but in Pakistan you go out more and walk more, the sun is out and you sweat and you have less health problems. [Participant 398, Pakistani, female, T2D]*

Apart from reporting having a healthier diet in the East, a majority of participants described how the hot weather provided more opportunities to sweat in the heat. Participants reported the belief that sweating (i.e., benefit of holidaying in a hot climate, rather than sweating due to physical activity/exertion) helped to eliminate excess sugar and impurities from the blood to improve diabetes control.

*P: The heat and sweat…when you sweat the sugar levels stays in control. (Participant 296, Pakistani, male, T2D)*

*P: When I go there (East), I sweat it out all my impurities, you're just sweating it out. (Community participant 26, Indian, female, T2D)*

The meaning of a holiday in the East for some participants was also to have a “break” from their medication for diabetes. For instance, some participants described stopping their diabetes medication or altering their medication regime whilst on holiday “back home.”

*I: Did you take all your medication with you?*

*P: I think I didn't need it*

*I: So you stopped taking it?*

*P: Yeah because I didn't need to take it because my sugar levels were in very good control. (Participant 296, Pakistani, male, T2D)*

*P: Every time I've been it's in July when it's hot and I like hot weather. Everybody keeps saying how I can cope with the heat but I like it…I never take any medicine when I am there…for 6 or 7 weeks that I am there, I never take. (Community participant 27, Pakistani, female, T2D)*

Compared to when they are in the UK, some participants strongly believed that their diabetes was cured or had disappeared whilst being “back home” in the East.

*P: When I go there my diabetes is gone…I feel good but when I come back it's gone higher. (Community participant 42, Pakistani, female, T2D)*

On return to the UK, participants described being less active due to poor weather conditions, especially in the winter. For some participants, the lack of exercise in the West was also related to poor mobility and health.

*P: I used to go walking but I've got knee problem and sometimes foot problem. I think these days because of the weather I feel worse…the doctor told me I need vitamin D and has given me tablets to take. [Participant 326, Pakistani, female]*

*P: I don't do enough exercise here (West) because the climate is different from Bangladesh errm…the glucose stays in the blood and the cholesterol is higher which it normally wouldn't be in Bangladesh…because I don't do any form of exercise (in the West). I don't sweat it off that's what I think. [Participant 5, Bangladeshi, male, T2D]*

There were tensions between participants having knowledge about the importance of exercise for diabetes and being self-aware of the little time they actually spent exercising in the UK and the effect this may be having on their diabetes.

A small number of participants with T1D described the difficulties of managing their diabetes when holidaying in the East. One of the main difficulties was travelling with insulin and not being able to store it at the correct temperatures.

*P: These days in Pakistan it's terrible conditions, no electricity for about 8 hours…so in the summer it's very difficult and because I take insulin I have nowhere to store it when the electricity goes. It's supposed to be stored between 2-8c and sometimes it can take up to 16 hours for the electricity to come back…the problem is the fridge won't work which means the efficacy of my insulin will reduce…and I get sick there. [Participant 313, Pakistani, male, T1D]*

For a small number of UK born South Asians (*n* = 4), adapting to the diet in the East was a strong concern and one participant in particular described being reluctant to try any of the food or drink tap water whilst on holiday in Pakistan due to fears of becoming ill.

*P: As far as the diet goes, it's nil and void, basically…when we got there, I bought a fridge…the water's not very good there, so I bought bottled water,…if you eat from there (Pakistan), your stomach is going to go so, basically, I just instructed my wife to get…you know, beans and get loads of potatoes and stuff like that and just ate chips and beans for a fortnight. [Participant 398, Pakistani, male, T1D]*

### 4.3. Limited Role Perceived for GPs/Practice

Participants described the role of the GP in supporting the management of diabetes as limited to prescribing medication and suggested they attended the GP only in response to invitation from the practice for routine check-ups and vaccinations.

*P: GP doesn't do anything just prescribes medication that's it. In all these years I hardly go to the doctors…I am on repeat prescription and my daughter rings the surgery and she just picks up the prescription. I just go for my vitamin or flu injections when they write to me. [Participant 393, Pakistani, male, T2D] *

*P: GP doesn't explain anything they just give medicine. [Participant 364, Nepalese, Male, T2D]*

There was a tension between having more frequent contact with the PN for their diabetes care and believing that the GP was the best person to seek information from for diabetes. Participants described receiving very little information and support about diabetes when they consulted their GP and suggested this may be due to the GP being pressured for time.

*P: The doctor just prescribes my medicines. [Participant 95, Indian, Female, T2D]*

*P: I don't get much advice from my GP, I just get my tablets and that's it (big laugh)…you can't blame them because they are seeing so many patients a day, they haven't got the time to spend 20 minutes or half an hour to talk and tell you things….people do listen to the GP, its coming from the horses' mouth you know….we rely on the GP for information. I mean I listen to my doctor. [Community participant 5, Indian, male, T2D]*

The limited access to information and support from the preferred source, which was the GP, resulted in GPs being perceived as having a limited role around prescribing and social networks including GPs aboard being at the forefront of new information related to diabetes.

*P: My family and friends can support me when there are new developments, like my cousin called me last week to inform me about new insulin which you only have to take once and told me to ask my GP…the GP doesn't have enough time but the nurse has more time and she is very helpful. My GP in Pakistan I talk to him and get advice over the phone about my diabetes. [Participant 313, Pakistani, male, T2D]*

This participant described help-seeking from GP whilst abroad, but few other participants reported this.

Overall, there seemed to be some disappointment and dissatisfaction expressed by some of the participants with regards to the care they received from their GP for their diabetes. Even those participants who expressed satisfaction with the care they received from the PN wanted more information and support from their GP for their diabetes.

## 5. Discussion

### 5.1. Summary

This is the first qualitative study in the UK to explore beliefs and practices of diabetes management in migrant British South Asians, whilst spending extended holidays in their native country. The main findings of this study show migrant British South Asians express a preference to be in a hot climate and change their diabetes management practices either by altering or abandoning their diabetes medication. The study findings also inform on the perceived role of the GP for diabetes care in the UK as being limited and the differences in the support received for diabetes management from social networks abroad in the East compared to the UK. The families abroad were an important source of information for diabetes, and their availability facilitated in participants taking up more exercise and eating a healthier diet until they return back to the UK.

### 5.2. Comparisons with Previous Literature

The existing literature reports on the impact of South Asians migrating from the East on factors such as genetics, diet, lifestyle, and psychological wellbeing, with implications for the onset and management of diabetes in the West. In the present study, participants' social context appeared to influence their beliefs about medication, as being back in a hot climate was believed to improve diabetes control or cure diabetes temporarily due to sweating in the heat. Studies conducted to assess knowledge and attitudes of diabetes with South Asian patients residing in the East have reported similar findings. In their Knowledge, Attitude, and Practice (KAP) survey with 238 diabetes patients, Shah et al. (2009) found that 63% had poor knowledge of diabetes and the importance of lifestyle modification, whilst 39% believed that diabetes could be cured. Low levels of literacy were also a common barrier to diabetes management [13]. Choudhury et al. (2014) [12] used a KAP survey to assess insulin use in 358 diabetes patients in tertiary care hospitals in India. Higher educational and socioeconomic status was associated with better understanding of insulin use and complications related to diabetes. Although a longer duration of diabetes was associated with better knowledge, 45% believed that food therapies (bitter gourd) could be used to control blood sugar levels.

The influence of beliefs and cultural practices has been shown to impede with diabetes management in this group [27]. However, in their study with British Bangladeshi men with diabetes, Greenhalgh et al. (1998) [28] showed that this group of men held strong beliefs about the benefits of sweating in the East for diabetes control and related the absence of sweating due to poor weather conditions in the West as one of the causes of diabetes. Our study findings extend on this work as the lack of adherence to medication whilst in the East suggests that participants in our study may have a poor understanding of the potential consequences of stopping or altering their medication for a long period of time. Other studies have shown the importance of personal models (*i.e., patient's beliefs about treatment effectiveness*) in diabetes [29, 30]; however, in the present study, the social context and location in which the participants manage their diabetes (i.e., in the company of family members with diabetes in the East) seemed to have a greater impact on treatment beliefs and self-management behaviours. In addition, participants appeared to have their own Explanatory Models (*i.e., interpretations of illness and treatment from different sources*) [31] of diabetes in the East which they seemed to carefully observe whilst on holiday, as well as drawing on the knowledge and practices of others (e.g., social networks) to make sense of their own diabetes in this social context. This context also seemed to provide an important lens through which participants chose to manage their diabetes whilst on holiday.

Research on the role of social networks has highlighted the importance of the support received from personal networks for illness management [18], particularly, the actions, practical, and emotional support that members of peoples' personal networks undertake [21]. For example, access to different types of network members has been found to provide access to a range of resources [32] and information [18]. However, the finding of our study extends the previous research and theorising about people being embedded in a “single” social network and the tensions between these, into a new area of “multiple networks” for diabetes management. The participants in our study appear to have two different and largely independent social networks, one in the East and one in the West, and their management behaviours, attitudes, and the support they receive differ between these networks. Whether people changed their self-management behaviours when in the East as a result of social network influences or a result of different opportunities (e.g., availability of fresh food and warmer weather) is unknown.

Other authors have also highlighted the importance of contextual influences in shaping individuals' health and wellbeing [33, 34], particularly in the South Asian group [19]. For example, the finding that participants make positive lifestyle changes such as walking and eating healthier foods with the family, compared to when they are in the UK, suggests that they were able to engage in self-care behaviours collectively with family members whilst in the East. However, participants struggled to engage in these behaviours independently when they returned back to the UK, with the climate, availability of fresh foods, and mobility being stated as barriers. The self-categorisation theory [35] provides a plausible interpretation for this finding in that the social context seemed to provide participants with motive and opportunities to compare their behaviour with others [35, 36]. Thus, the way in which participants perceived themselves in the East and the West seemed to have implications for both diabetes-related beliefs and management behaviours. In addition, the tensions between the dissatisfaction of the care received from their GP (UK) for their diabetes may explain why participants turn to their social networks for support and information [18, 27].

### 5.3. Strengths and Limitations

The analysis was undertaken in an interdisciplinary team (with expertise in psychology, health services research, and primary care), which increases trustworthiness of the analysis [37]. The interview guide contained a range of topics related to diabetes management and social networks and holidaying in the East was one of a number of topics explored.

There are limitations to the present study. Although recruitment took place in several areas of Greater Manchester to target an adult population from various subethnic South Asian groups, backgrounds, and age to increase sample variety, most participants were first generation immigrants, from deprived communities, with T2D, whose first language was not English, and some were illiterate in their native language. The migrant status may have also been a key factor in shaping the knowledge, belief, and attitudes towards diabetes. Therefore, it can be argued that this sample may not sufficiently reflect the more-educated sections of the South Asian community, British born South Asians, and patients with T1D as 90% of the sample had T2D, and we believe that our findings primarily relate to this group. We did not collect specific information on socioeconomic status. However, T2D remains as a significant problem in South Asian people from lower socioeconomic backgrounds and warrants research. A professional interpreter, Diabetes Asian Link worker, and the participants' family members facilitated some of the interviews. This may have influenced the data in that the interviewees' responses may not have been captured accurately, as the interpreters may have found it easier to summarise the respondents' answers to the questions asked, rather than interpret each answer in verbatim [38].

### 5.4. Implications for Policy and Practice

The social context and the support received from social networks whilst holidaying in the East had an influence on beliefs and behaviours related to diabetes management. This suggests that this patient group appear to have a poor understanding of the importance of adhering to diabetes medication when holidaying in the East. Culturally tailored, community-based diabetes management programmes may facilitate and increase motivation to engage in a healthy lifestyle and better manage diabetes on return to the UK [39].

Current policy guidelines on diabetes management do not inform on pretravel advice/education for patients or provide guidance on altering patients' diabetes medication during travel, apart from the importance of adhering to medication for positive clinical and health outcomes [40]. Of the limited information available on travel and diabetes, patients are advised to seek care and information on diet and medication before travel [23, 41] to minimize fluctuations in glucose control and reduce other travel related risks [42]. Of the few studies available on diabetes management during travel, most are on travel-related problems in people with T1D [24, 43]. Given that the migrant British South Asians in this study indicated a high regard for holidaying in the East, tailored pretravel education for patients and their social networks may inform them on the importance of diabetes management and seeking pretravel advice before going to the East. Health care practitioners in primary care may also benefit from training and skills into the beliefs held about diabetes in migrant British South Asians and the changes independently made to their diabetes regimen in order to help improve adherence to medication whilst on holiday and reduce potential future complications and healthcare costs including medication wastage and mortality.

## 6. Conclusion

Holidaying in the East is an important social and cultural tradition for the migrant British South Asian population. The availability of social networks in the East and the information received on diabetes (diet and exercise) from networks seemed to be valued and resulted in participants engaging in a healthier lifestyle during their stay. However, the informed decision to refrain and/or alter their diabetes medication due to the belief that diabetes disappears in the East, as a result in the change in climate providing the opportunity to sweat and eliminate excess sugar from the body, suggests that some migrant British South Asians have a poor understanding of diabetes and the importance of adhering to medication.

Future research needs to explore whether patients are seeking professional advice on how to manage their diabetes whilst on an extended holiday. This will help to inform pre-travel diabetes education resources for patients and their social networks to reduce potential future complications of diabetes and healthcare costs to the NHS.

## Figures and Tables

**Table 1:** Demographics.

	*n* (%)
	**44 **
Male	23 (52)
Female	21 (48)
Age, years (SD = 12.5, range)	61 (32–84)
Diabetes	
T1D	5 (11)
T2D (*n* = 7 on insulin)	39 (91)
Duration of diabetes	
0 to 5 years	11 (25)
5 to 10 years	16 (36)
10 years+	17 (39)
Marital status	
Married or in civil partnership	38 (86)
Other	6 (14)
Subethnic groups	
Indian	22 (50)
Pakistani	18 (41)
Bangladeshi	3 (7)
Other (Nepalese)	1 (2)
Education	
No qualifications	19 (44)
1 to 4 O levels	3 (7)
A levels	1 (2)
Other qualifications	4 (9)
NVQ	3 (7)
Professional qualifications	5 (11)
First degree	5 (11)
Higher degree	4 (9)
Born in the UK or migrated to UK	
British born South Asians	4 (9)
British migrant South Asians	40 (91)
